# The Prevalence of Metabolic Syndrome According to Grip Strength in Teenagers

**DOI:** 10.3390/children8020108

**Published:** 2021-02-04

**Authors:** Duk Han Ko, Young Kyun Kim

**Affiliations:** 1 Department of Sports Science Convergence, Dongguk University, Seoul 04620, Korea; kodh119@hanmail.net; 2 Graduate School of Sports Medicine, CHA University, Seongnam 13496, Korea

**Keywords:** children, adolescents, metabolic syndrome, grip strength

## Abstract

The prevalence of metabolic syndrome in adolescents is increasing. Recently, the relevance of grip strength as a factor of metabolic syndrome in this population has raised questions. This study investigated the prevalence of metabolic syndrome according to grip strength in children and adolescents using large-scale data from the Korean National Health and Nutrition Survey (KNHNS). From 2014 to 2018, 1527 boys and 1292 girls participated in the KNHNS. The participants were classified into three groups according to age: 10–12 years (early teenager, ET), 13–15 years (middle teenager, MT), and 16–18 years (late teenager, LT). The participants were classified as having metabolic syndrome if they fulfilled three of the adolescent metabolic syndrome criteria. The grip strength was divided into groups with high and low grip strength, respectively, and the cutoff value for the prevalence was calculated using receiver operating characteristic curve analysis. There were significant differences in waist circumference, high-density lipoprotein cholesterol, and triglyceride levels based on grip strength in the ET, MT, and LT groups. Therefore, the prevalence of metabolic syndrome was lower when grip strength was higher. The cut-off values of the relative grip strength (kg/body weight) to predict metabolic syndrome among boys were 0.349, 0.466, and 0.485 for the ET, MT, and LT groups, respectively. The corresponding cut-off values for girls were 0.373, 0.383, and 0.382, respectively. In conclusion, there is a non-linear relationship between grip strength and metabolic syndrome in adolescents.

## 1. Introduction

Metabolic syndrome (MetS) refers to abnormal metabolic function and includes factors such as high blood pressure (BP), diabetes, low high-density lipoprotein cholesterol (HDL-C), and high triglyceride (TG) levels. MetS is closely related to the occurrence of cardiovascular disease [1]. The incidence of MetS is increasing worldwide. Furthermore, the age at onset is decreasing, and it has become common among adolescents [2,3]. The incidence of MetS in South Korea is reported to be 20–30% in adults and 3.9–7.1% in adolescents [4,5]. MetS is a major problem in adolescents because the MetS that occurs during childhood and adolescence often persists in adulthood [6].

The main causes of MetS are low physical activity, obesity, and low muscle mass and strength accompanied by high caloric intake [7,8,9]. These are also the causes of MetS in adolescents; these individuals have decreased physical activity and therefore decreased physical strength [4,10]. The method used worldwide to test physical strength is the grip strength test, which is a safe and simple measurement method [11]. Previous studies have examined the relationship between low grip strength and MetS and found that low grip strength is associated with diabetes, cardiovascular disease, mortality, and MetS [12,13]. Another study examined grip strength in patients with chronic diseases such as high BP and diabetes and found that grip strength is significantly lower among patients with these chronic diseases, even after correcting for variables such as race, age, sex, and smoking [14]. However, the association between MetS and grip strength in adolescents has been poorly researched. Grip strength is the most common test method for representing muscle strength, and its relationship with many diseases has already been proven. Adolescence is also a transitional stage from childhood to adulthood, and there is rapid physical development and physique development during this period [15,16]. Therefore, the purpose of this study was to investigate the relationship between relative grip strength and MetS in children and adolescents. A cross-sectional study was conducted to determine the baseline values for MetS and relative grip strength by dividing children and adolescents into three age groups. The hypothesis of this study is that children and adolescents with MetS will have low grip strength due to lack of physical activity.

## 2. Methods

### 2.1. Participants

**Figure 1:**
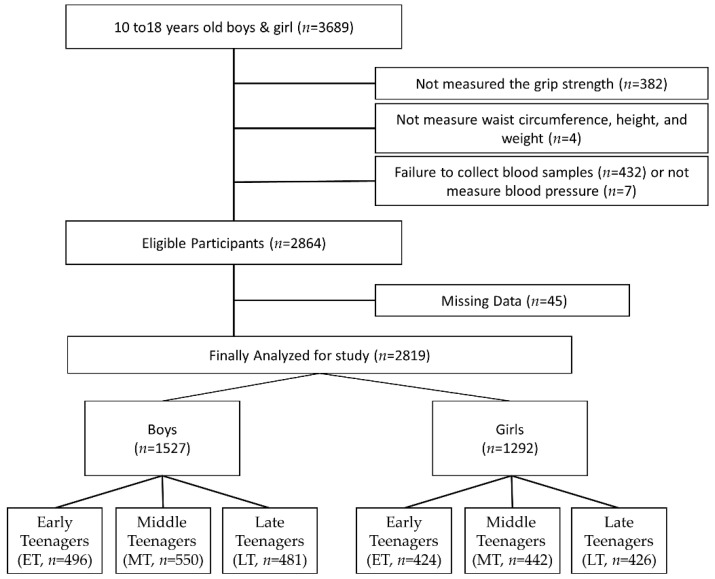
Diagram of participant inclusion and exclusion. Abbreviations: ET, early teenager; MT, middle teenager; LT, late teenager.

To investigate the association between MetS and grip strength by age in children and adolescents, 3689 people aged 10–18 years who participated in the Korea National Health and Nutrition Survey (KNHNS), 2014–2018, were included in this study. The participants were physically healthy elementary to high school students, and data were collected from all regions of Korea. KNHNS is a health dataset supervised by the Korea Centers for Disease Control and Prevention, and it checks the health status of participants by collating the current status and trend of the people’s health and nutrition according to the National Health Promotion Act. It is an important tool for evaluating health outcomes in this population. The inclusion criteria for this study were participants who underwent testing for all factors related to MetS, underwent grip strength measurement, and agreed with both specimen and data collection for this study. Those who did not perform grip strength due to hand or arm injury or other reasons (*n* = 382), did not measure waist circumference, height, and weight (*n* = 4), and could not obtain blood samples (*n* = 432) were excluded from this study. In addition, those with missing data (*n* = 45) that cannot be used for data such as 0 were also excluded. Therefore, the data of 2819 participants (1527 boys and 1292 girls) were analyzed. The participants were classified into three groups by age: individuals 10–12 years old were classified as early teenagers (ET), those 13–15 years old as middle teenagers (MT), and those 16–18 years old as late teenagers (LT). To comply with research ethics, the purpose of the test and the use of the results for research purposes were explained, and the data of those who agreed were used. This study was approved by the research ethics committee of the affiliated institution (2015-01-02-6C). Figure 1 describes the diagram of this study.

### 2.2. Grip Strength Test

Grip strength was measured using a grip strength dynamometer (TKK 5401, TAKEI, Niigata, Japan). The test method and procedure were performed according to the American College of Sports Medicine test method [17]. The participants were dressed in comfortable clothing. The inspector explained the sufficient warm-up and inspection methods before the test and performed a demonstration to help the participants understand. Then, the second joint of the participant’s finger was adjusted to fit the handle. The participant stood with a straight waist and looked straight ahead. The arms were abducted by about 30 degrees to prevent them from touching the thighs, and the elbows were not bent. At the tester’s signal, the participant held the grip of the grip dynamometer with the strongest possible force for 2 s. The posture was not disturbed, and the participant was instructed not to bend the elbows or waist or lower the head. The grip strength test was performed first on the left side three times and then on the right side three times, and the best value was recorded. The measurement was recorded in kg, and the measured value for analysis was taken as the absolute value. The absolute value was divided by the body weight of the participant to yield the relative value.

### 2.3. Metabolic Syndrome Diagnosis

This study used the criteria proposed by Cook et al. [6] to diagnose MetS. MetS was diagnosed when three or more of the following criteria were fulfilled: waist circumference ≥ 90th percentile, BP ≥ 90th percentile, TG ≥ 110 mg/dL, HDL-C ≤ 40 mg/dL, fasting blood glucose ≥ 110 mg/dL, and prescription of blood pressure medication, hyperlipidemia medication, or diabetes medication.

#### 2.3.1. Waist Circumference

The participants stood with the feet 25–30 cm apart and comfortably maintained breathing while the examiner measured the waist circumference. The thickest part of the lowest position of the ribs and the highest position of the pelvis (iliac crest) were measured to 0.1 cm precision with a tape measure not pressing the skin [18].

#### 2.3.2. Blood Pressure

BP measurements were performed using a mercury sphygmomanometer according to standard guidelines [19]. The participants rested for 5 min. Then, the lower part of the compression cuff was placed 3 cm above the elbow and closed securely. The size was chosen to ensure that the cuff could cover 2/3 of the upper arm and that the middle part of the cuff was on top of the brachial artery. With the stethoscope placed, the cuff was inflated to the maximum inflation pressure at a rapid and constant rate. The valve was adjusted so that the pressure dropped constantly at a rate of 2 mm Hg per second. The reading where the Korotkoff sound first occurred was classified as the systolic BP, and the last reading before the disappearance of the Korotkoff sound was classified as the diastolic BP.

#### 2.3.3. Blood Collection

The Participants fasted for at least 8 h before blood collection, and excessive physical activity was prohibited until measurement. Blood was collected mainly through the median cubital vein, and the total amount of blood collected was about 25–30 mL [20]. The variables measured through blood collection were biochemical properties related to metabolic diseases, such as fasting glucose, TG, and HDL-C.

### 2.4. Data Analysis

SPSS 25.0 (SPSS Inc., Chicago, IL, USA) was used for statistical analyses. The data did not form a normal distribution as a result of performing a normality test using Kolmogorov–Smirnov (*p* < 0.05). Therefore, the non-parametric statistics method was applied to compare the variables of the continuous variables in Table 1 and Table 2. General characteristics and metabolic syndrome variables are expressed as mean and standard deviation. For continuous variables, non-parametric Kruskall Wallis test was performed to test the difference between the age groups. The non-parametric Wilcoxon Mann–Whitney test was conducted to determine whether there was a significant difference in MetS factors based on the mean grip strength. The prevalence of MetS according to grip strength was evaluated using the chi-square test. The cut-off value of grip strength to predict MetS was calculated using receiver operating characteristic curve analysis (MedCalc; MedCalc Ltd., Ostend, Belgium). This software presents the optimal cut-off values using specificity and sensitivity and shows the area under the curve as a result. In this study, the age-adjusted odds ratio (OR) was calculated by dividing the participants into two groups based on the presented cut-off value and performing logistic regression analysis. The significance level was set at *p* < 0.05. This study was conducted as a cross-sectional design study.

## 3. Results

### 3.1. General Characteristics

Table 1 shows the general characteristics of the participants. The patients were classified according to sex and age, and there were significant differences in height, weight, body mass index (BMI), absolute grip strength, and relative grip strength between the age groups.

### 3.2. Cut-Off Value for Grip Strength to Predict MetS

**Figure 2:**
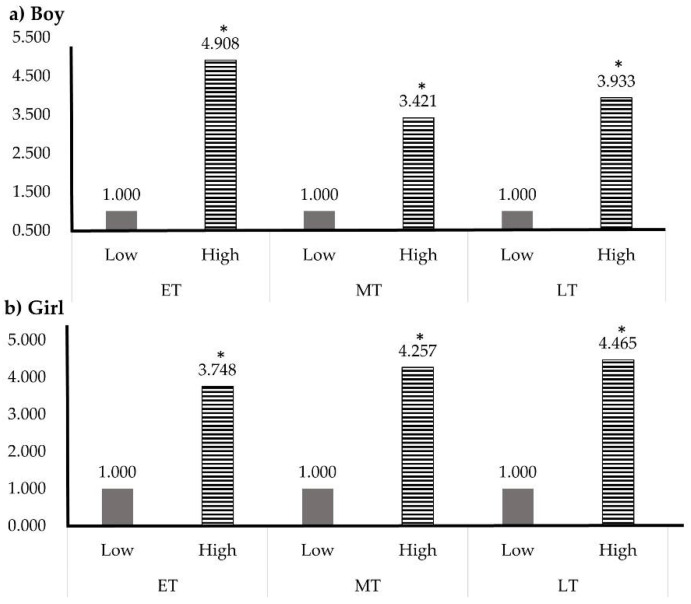
Odds ratios for metabolic syndrome according to relative grip strength. (**a**), OR for metabolic syndrome according to the relative grip strength of boys. (**b**), OR for metabolic syndrome according to the relative grip strength of girls. * *p* < 0.05. Abbreviations: OR, odds ratio; ET, early teenager; MT, middle teenager; LT, late teenager.

Table 2 shows the grip strength cut-off values that predict MetS. The cut-off values (kg/weight) of the relative grip strength for MetS in the ET group were 0.349 (AUC 0.865, 95% CI 0.767–0.839, *p* = 0.031) and 0.373 (AUC 0.751, 95% CI 0.715–0.788, *p* = 0.043) in boys and girls, respectively, and the prevalence of MetS in the low-grip strength group increased 4.908 times in boys and 3.748 times in girls. The cut-off values of the relative grip strength in the MT group were 0.466 (AUC 0.777, 95% CI 0.732–0.804, *p* = 0.032) and 0.383 (AUC 0.753, 95% CI 0.711–0.792, *p* = 0.047) for boys and girls, respectively. The incidence of MetS increased 3.421 times in boys with low grip strength and 4.257 times in girls with low grip strength. The cut-off values of relative grip strength for MetS in the LT group were 0.485 (AUC 0.825, 95% CI 0.789–0.858, *p* = 0.029) for boys and 0.382 (AUC 0.778, 95% CI 0.737–0.816, *p* = 0.040) for girls. The prevalence of MetS in adolescents with low grip strength increased 3.933 times in boys and 4.465 times in girls. Figure 2 shows the ORs of MetS according to the relative grip strength by sex and age.

### 3.3. Association between Grip Strength and MetS

The characteristics of MetS factors in participants with high and low grip strength are shown in Table 3. In the ET group, there were significant differences between individuals with high and low grip strength in waist circumference, HDL-C, and TG in both boys and girls. In the MT group, both boys and girls showed significant differences in waist circumference, HDL-C, and TG between individuals with high and low grip strength, and there were significant differences in blood glucose levels based on grip strength among the female participants. In the LT group, there were significant differences in waist circumference, HDL-C, and TG according to grip strength, and among male participants, there was a significant difference in systolic BP based on grip strength. The incidence of MetS based on grip strength is shown in Table 4. There was a significant difference in the incidence of MetS according to low and high grip strength in all age groups among both boys and girls. In particular, the statistical significance of the MT and LT groups was greater than that of the ET group.

## 4. Discussion

The purpose of this study was to investigate the relationship between grip strength and the prevalence of MetS in adolescents according to age and sex. This study selected and analyzed grip strength as a predictive factor. Grip strength analysis is a tool to evaluate upper body strength and is the most economical and simple strength test [11]. The grip force, which is the force exerted when an object is grasped or pulled, is commonly used as a measure of the function and disorder of the musculoskeletal system [16]. In addition, since the risk to the body is low and the test can be used by participants of any age, many previous studies have used the grip strength test as the most representative test to classify health and physical conditions [21,22]. Since grip strength may differ according to an individual’s weight and height, the relative grip strength value, which is the measured grip strength divided by the weight, is preferable [22,23]. This study classified adolescents into age groups to investigate the association between grip strength and MetS. Both the absolute and relative grip strengths were analyzed. This is particularly important in adolescents as their weight changes rapidly, and analyzing only the absolute grip strength can limit the generalizability of the study [24,25]. It is difficult to generalize the absolute gripping force value because growth proceeds rapidly in this population.

In this study, indicators related to MetS were significantly different between adolescents with high grip strength and adolescents with low grip strength. Specifically, this phenomenon was observed with regard to waist circumference, HDL-C, and TG in both boys and girls. This is likely the effect of exercise and physical activity. Exercise and physical activity have a positive effect on waist circumference, HDL-C, and TG in adolescence [25]. Lopes et al. reported in a meta-analysis that moderate- to high-intensity continuous exercise, resistance exercise, and high-intensity interval training (HIIT) exercise in adolescents helped increase HDL-C [26]. Chen et al. followed 25 obese adolescents aged 16–18 years who performed aerobic exercise for 8 weeks. The waist circumference decreased from 94.2 cm (standard deviation [SD] = 1.59) before the experiment to 88.5 cm (SD = 2.02) after the experiment, which was a statistically significant decrease compared with that of the control group [27]. Whooten et al. found that exercise is useful for reducing TG levels in adolescents. In particular, they reported that HIIT exercise is more effective in reducing TG levels than medium- to high-intensity continuous exercise [28]. In addition, adolescents with high physical activity have a much lower prevalence of MetS, which is associated with lower insulin resistance [28]. It has also been reported that people with limited physical activity due to disease have a high prevalence of MetS [29]. In addition, in Cho’s study of 931 adolescents with an average age of 13.8 years, the prevalence of adolescent MetS was 3.1% for boys and 0.8% for girls, and 95% of participants with MetS were obese [30]. This means that the prevention of obesity through physical activity in adolescents acts as a preventive factor for MetS.

We observed significant differences in the prevalence of MetS according to grip strength. Previous studies have investigated the relationship between the relative grip strength and MetS in adult men and found that a higher relative grip strength was associated with a lower risk of MetS [3,31]. Similar results in adolescents were observed in this study. We classified the age of adolescents by dividing them into three categories. In the ET group, there was a nonlinear relationship between the prevalence of MetS and grip strength in both males and females. In addition, the ET group had significantly lower grip strength than the MT and LT groups. This is also likely a result of exercise and physical activity. After age 13, when musculoskeletal growth begins, an increase in grip strength through appropriate exercise can have a positive effect on development, which can directly affect the prevalence of MetS [32]. In Moon et al.’s study on MetS in adolescents, the prevalence of MetS in adolescents with a waist circumference > 90 cm for boys and >85 cm for girls was 9.2%. The prevalence was 7.3% when the TG level was >150 mg/dL, and the prevalence was 9.9% when the fasting blood glucose level was >100 mg/dL. In particular, the prevalence of MetS was 24.4% when the HDL-C level was <40 mg/dL for boys and <50 mg/dL for girls [33]. However, these risk factors can be managed and prevented through exercise. In Dencker’s study, a negative correlation between body fat mass and physical activity was reported in 248 children aged 8–11 years. In addition, obese children averaged 12 fewer minutes of high-intensity physical activity per day than children with a normal BMI [34]. Therefore, their results recommend appropriate physical activity for the prevention of MetS. Haider’s study established a link between grip strength and MetS. Therefore, various physical activities are recommended as a method to increase grip and prevent MetS [35]. In addition, Gomes et al. reported that proper physical activity is the most effective alternative to not only improve grip strength but also lower the prevalence of MetS [36]. According to Park’s study, the grip strength of adolescents who performed regular aerobic exercise and strength exercise more than twice a week was significantly higher than that of adolescents who did not. The prevalence of MetS among adolescents in the top 25% of relative grip strength was 1.1%, and the prevalence of MetS in adolescents in the lower 25% was 9.6%, showing a significant difference [15]. In this study, the prevalence of MetS was investigated according to age and relative grip strength in adolescents. In all age groups, regardless of gender, high grip strength was associated with a lower incidence of MetS than low grip strength. As shown in Figure 1, in the case of males in the ET group, the OR for adolescents with low relative grip strength was 4.908 compared to adolescents with high relative grip strength. In addition, the ORs of MetS in males with low grip strength in the MT and LT groups were 3.421 and 3.933, respectively. Females with low grip strength also had an increased risk of MetS, with ORs of 3.748, 4.257, and 4.465 in the ET, MT, and LT groups, respectively. Therefore, adolescents with low relative grip strength were more likely to develop MetS.

One of the unique findings of this study is that we identified the cut-off value of grip strength to predict MetS. Adolescents with high grip strength showed a low prevalence of MetS. In a similar study, Bang et al. showed that the ORs for MetS in inactive adolescents compared to adolescents who regularly performed physical activity were 1.115 for boys and 1.198 for girls [37]. Garcia-Hermoso et al. assessed cut-off values for grip strength and risk factors for MetS in 1795 adult men and women aged 18 to 30 years and found that the cut-off value of grip strength for predicting MetS in men was 0.466, and it was 0.332 in women [38].

It is necessary to take measures to prevent adolescents from developing MetS because the time to onset of chronic diseases can be accelerated in their 20 s to 30 s and can cause many health problems. In particular, early management of childhood obesity has been suggested as a way to prevent metabolic syndrome after growing age [15,39,40].

There are relatively few studies on the relationship between grip strength and MetS in adolescents; therefore, this study is rare. However, it has several limitations. This study limited the examination of physical strength to grip strength. A previous study found that examining lower extremity muscle strength using isokinetic equipment and lower teeth resulted in a stronger association with MetS, although that study examined only elderly participants [8]. While other studies have shown that cardiopulmonary endurance and MetS are significantly related, no significant relationship was found between MetS and grip strength [41]. Therefore, it is necessary to investigate the relationship between various physical factors and MetS in adolescents. In addition, it was impossible to control the participants’ daily lives during the examination period. Lastly, although the relative grip strength was used, the participants’ muscle mass and fat mass were not considered. Future studies that supplement these limitations should be conducted.

## 5. Conclusions

This study classified adolescents into three age groups and studied the prevalence of MetS according to grip strength. The prevalence of MetS was low when grip strength was high. In particular, waist circumference, HDL-C, and TG levels showed statistically significant differences according to grip strength among male and female adolescents in all three age groups. In addition, the blood glucose level of MT girls also showed a significant difference based on grip strength, and the systolic BP of LT boys was significantly different based on grip strength. Education on risk factor management and appropriate physical activity are recommended for the prevention and management of MetS in teenagers.

## Figures and Tables

**Table 1:** Characteristics of participants.

Variables	Boy			Girl		
	ET (*n* = 496)	MT (*n* = 550)	LT (*n* = 481)	ET *n* = 424)	MT (*n* = 442)	LT (*n* = 426)
Age, year	11.0 (10.0–12.0)	14.0 (13.0–15.0) ^a^	17.0 (16.0–18.0) ^b,c^	11.0 (10.0–12.0)	14.0 (13.0–15.0) ^a^	17.0 (16.0–18.0) ^b,c^
Height, cm	150.0 (144.1–156.2)	169.0 (165.0–173.4) ^a^	173.4 (169.6–177.9) ^b,c^	151.0 (144.9–156.0)	159.8 (156.2–163.1) ^a^	161.2 (157.8–164.8) ^b,c^
Weight, kg	44.1 (36.5–52.3)	60.0 (52.5–69.2) ^a^	65.5 (58.1–75.0) ^b,c^	41.7 (35.9–48.4)	51.2 (46.2–57.7) ^a^	55.1 (50.0–62.0) ^b,c^
BMI, kg/m^2^	19.2 (17.1–22.3)	20.6 (18.5–23.9) ^a^	21.7 (19.6–24.5) ^b,c^	18.4 (16.7–20.6)	20.2 (18.4–22.4) ^a^	21.2 (19.5–23.7) ^b,c^
Grip, kg	17.5 (15.0–21.5)	31.0 (26.5–36) ^a^	36.5 (32.5–41) ^b,c^	16.0 (13.5–19.5)	21.5 (19.0–24.5) ^a^	23.5 (20.5–26.5) ^b,c^
Grip, kg/BW	0.41 (0.35–0.47)	0.52 (0.44–0.59) ^a^	0.56 (0.48–0.63) ^b,c^	0.40 (0.33–0.45)	0.42 (0.37–0.47) ^a^	0.45 (0.39–0.51) ^b,c^

The values are expressed as median (percentile 25th–75th); a, ET vs. MT; b, ET vs. LT; c, MT vs. LT. Abbreviations: ET, early teenager; MT, middle teenager; LT, late teenager; BMI, body mass index; BW, body weight.

**Table 2:** Receiver operating characteristic curve analysis of relative grip strength.

Group	Cut-Off	AUC (95% CI)	Sensitivity	Specificity	*p*
Boy					
ET	0.349	0.865 (0.767–0.839)	88.2	78.0	0.031 *
MT	0.466	0.777 (0.732–0.804)	69.2	69.9	0.032 *
LT	0.485	0.825 (0.789–0.858)	80.6	77.8	0.029 *
Girl					
ET	0.373	0.751 (0.715–0.788)	85.7	60.4	0.043 *
MT	0.383	0.753 (0.711–0.792)	66.7	74.2	0.047 *
LT	0.382	0.778 (0.737–0.816)	81.8	67.8	0.040 *

* *p* < 0.05. Abbreviations: AUC, area under the curve; CI, confidence interval; ET, early teenager; MT, middle teenager; LT, late teenager; MetS, metabolic syndrome.

**Table 3:** Risk factors of metabolic syndrome according to grip strength levels.

Variables	Boy			Girl		
	Grip Low	Grip High	*p*	Grip Low	Grip High	*p*
ET						
Waist C., cm	71.7 (63.7–78.4)	62.5 (58.2–67.6)	<0.001 *	65.9 (61–72.2)	60.5 (56.5–65)	<0.001 *
SBP, mmHg	108 (100–114)	105 (99–113)	0.097	104 (99–111)	104 (99–111)	0.811
DBP, mmHg	62 (58–68)	63 (57–69)	0.171	63 (59–68)	63 (58–68)	0.541
HDL-C, mg/dL	51 (45–59)	54 (47–62)	0.005 *	50 (44–57)	53 (46–61)	0.005 *
TG, mg/dL	75 (51–112)	64 (44–88)	0.033 *	93 (64–131)	77 (54–105)	0.006 *
Glucose, mg/dL	95 (91–98)	94 (90–98)	0.078	92 (89–96)	92 (88–96)	0.256
MT						
Waist C., cm	77.6 (69.7–86.1)	68.2 (64.3–73.2)	<0.001 *	70 (64.4–74.3)	64.6 (61.2–68.4)	<0.001 *
SBP, mmHg	110 (105–118)	110 (103–116)	0.145	106 (99–112)	105 (100–112)	0.853
DBP, mmHg	65 (59–72)	66 (61–72)	0.200	66 (61–72)	66 (61–71)	0.843
HDL-C, mg/dL	46 (41–54)	50 (44–56)	0.002 *	50 (44–57)	53 (47–60)	<0.001 *
TG, mg/dL	78 (59–114)	63 (46–87)	<0.001 *	83 (57–109)	72 (51–92)	0.003 *
Glucose, mg/dL	93 (89–97)	92 (88–96)	0.211	90 (86–95)	89 (85–93)	<0.001 *
LT						
Waist C., cm	81.3 (73.7–90.2)	70.8 (67.1–75.2)	<0.001 *	72.3 (67.3–80)	66.6 (63.6–70.7)	<0.001 *
SBP, mmHg	115 (108–123)	112 (105–118)	<0.001 *	106 (102–113)	105 (100–111)	0.074
DBP, mmHg	71 (66–76)	72 (66–75)	0.644	68 (63–74)	68 (63–72)	0.059
HDL-C, mg/dL	46 (41–53)	49 (42–55)	0.014 *	51 (46–58)	54 (48–60)	0.034 *
TG, mg/dL	84 (62–116)	70 (53–101)	0.003 *	78 (57–105)	70 (51–93)	0.021 *
Glucose, mg/dL	91 (88–96)	90 (86–95)	0.282	88 (85–93)	88 (83–92)	0.077

* *p* < 0.05 by Wilcoxon Mann–Whitney test; the values are expressed as median (percentile 25th–75th). Abbreviations: ET, early teenager; MT, middle teenager; LT, late teenager; Waist C, Waist circumference; SBP, systolic blood pressure; DBP, diastolic blood pressure; HDL-C, high-density lipoprotein cholesterol; TG, triglycerides.

**Table 4:** Metabolic syndrome prevalence and grip strength.

Group	Grip	Boy			Girl		
		Non-MetS	MetS	*p*	Non-MetS	MetS	*p*
ET	Low	247 (94.6%)	14 (5.4%)	0.005 *	205 (97.6%)	5 (2.4%)	0.007 *
	High	233 (99.1%)	2 (0.9%)		218 (99.1%)	2 (0.9%)	
MT	Low	241 (88.0%)	33 (12.0%)	<0.001 *	222 (93.3%)	16 (6.7%)	<0.001 *
	High	270 (97.8%)	6 (2.2%)		220 (99.1%)	2 (0.9%)	
LT	Low	205 (88.0%)	28 (12.0%)	<0.001 *	209 (91.7%)	19 (8.3%)	<0.001 *
	High	245 (98.8%)	3 (1.2%)		217 (98.6%)	3 (1.4%)	
*p* for trend		0.028 *			0.008 *		

* *p* < 0.05 by chi-square test; the values were expressed as *n* (%). Abbreviations: ET, early teenager; MT, middle teenager; LT, late teenager; Non-MetS, non-metabolic syndrome; MetS, metabolic syndrome.

## Data Availability

Publicly available datasets were analyzed in this study. This data can be found here: [https://knhanes.cdc.go.kr].
